# Photocatalytic reduction of aqueous chromium(vi) by RuO_2_/g-C_3_N_4_ composite under visible light irradiation†

**DOI:** 10.1039/d5ra00883b

**Published:** 2025-05-20

**Authors:** Yongjun Liu, Xiaohe Du, Zhiming Huang

**Affiliations:** a College of Environmental Science & Engineering, Dalian Maritime University Dalian 116026 P. R. China lyjglow@dlmu.edu.cn +86-411-84727670 +86-411-84725275

## Abstract

Graphitic carbon nitride (g-C_3_N_4_) has been extensively investigated as a novel nonmetallic visible-light response photocatalyst. However, its uses in photocatalytic reductions were limited because of the sluggish oxygen evolution reaction (OER) and the resulting self-decomposition. In this paper, a ruthenium dioxide loaded g-C_3_N_4_ composite (RuO_2_/g-C_3_N_4_) was prepared by forced oxidative hydrolysis of ruthenium(iii) chloride on the surface of g-C_3_N_4_ that was obtained by direct condensation polymerization of melamine. Photocatalytic reduction of aqueous Cr(vi) by it under illumination from a 400–410 nm light-emitting diode was examined. It was shown that the Cr(vi) reduction rate was much higher in RuO_2_/g-C_3_N_4_ than in pure g-C_3_N_4_. Without any sacrificial electron donor and at initial solution pH 2.3, Cr(vi) removal (200 mL and 0.5 mM) was 34% and 76.4% with 0.1 g pure g-C_3_N_4_ and 0.1 RuO_2_ (1.0 wt%)/g-C_3_N_4_, respectively. The optimum initial solution pH was 2.4. Methanol accelerates while acetone suppresses the Cr(vi) reduction significantly. Ferric ions catalyze the reduction, especially in the later stage. UV-Vis diffusion reflectance spectroscopy and theoretical analysis showed that RuO_2_ not only boosts the charge separation but also protects the g-C_3_N_4_ from decomposition by its extraordinary catalytic action for OER. The used RuO_2_/g-C_3_N_4_ was separated from the solution by microfiltration, with little leaching and residue remaining in the filtrate. The reclaimed RuO_2_/g-C_3_N_4_ was recycled for 5 cycles and no obvious decrease in catalytic activity was observed, indicating its superior potential in industrial applications.

## Abstract 2

Graphitic carbon nitride (g-C_3_N_4_) has been extensively investigated as a novel nonmetallic visible-light response photocatalyst.

## Graphical abstract

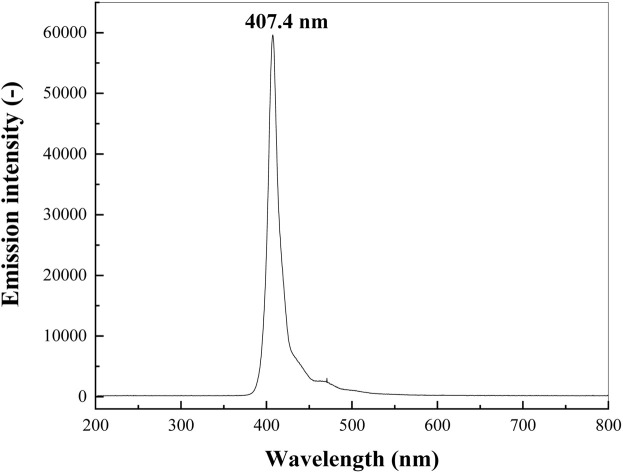

## 1. Introduction

Chromium is a common pollutant in water, where it usually exists in the extremely toxic and carcinogenic hexavalent [Cr(vi)] form.^1^ Converting Cr(vi) to much less toxic and precipitable trivalent form [Cr(iii)] *via* chemical reduction is a key step to treat the Cr(vi) contaminated wastewater. However, the chemical reduction needs excess reducing agents and acids to drive the reaction, adding tremendous amounts of neutralizing reagents in the subsequent precipitation.^2,3^ Therefore, developing atom-economic processes for Cr(vi) reduction is in great need.

Recently, graphite carbon nitride (g-C_3_N_4_) has emerged as a novel nonmetallic photocatalyst due to its narrow band gap (2.7 eV), visible light response capability, rich sources of precursors and simple preparation method.^4^ In theory, g-C_3_N_4_ can be applied for reduction of heavy metal ions because of its highly active conduction band electrons (*E*_cb_ = −1.1 eV *vs.* NHE, slightly dependent on fabrication method and reaction procedure). However, pure g-C_3_N_4_ exhibits low photocatalytic activity due to its high recombination speed of photo-generated charge carriers and its low conductivity. Constructing g-C_3_N_4_-based composites is considered as a promising strategy in promoting photocatalytic reductions. Wang *et al.*^5^ found that the 1D black phosphorus-tubular g-C_3_N_4_ can remove 94.1% of Cr(vi) with a rate constant of 0.0404 min^−1^. Ren *et al.*^6^ constructed a g-C_3_N_4_/NH_2_-UiO-66(Zr) heterojuncter by solvothermal and *in situ* deposition to effect both Cr(vi) reduction and tetracycline hydrochloride oxidation in aqueous solution, where the photocatalytic removal of Cr(vi) by CU-20 wt% forming heterojunction was 1.86 times that of pure NH_2_-UiO-66(Zr) under visible light irradiation. Eslamlu *et al.*^7^ reported that Sb_2_MoO_6_ coupled with g-C_3_N_4_ nano-tubes showed Cr(vi) reduction efficiency of 22 times higher than the bare g-C_3_N_4_. When MoS_2_/g-C_3_N_4_ was grafted with cyclodextrins, Cr(vi) reduction in the simulated agricultural wastewater was remarkably enhanced.^8^ Mohamed *et al.*^9^ synthetized mesoporous BiVO_4_/2D-g-C_3_N_4_ heterostructures for superior visible light-driven photocatalytic reduction of Hg(ii) in the presence of HCOOH.

Metal oxides or metal sulfides were usually employed to couple with g-C_3_N_4_ to improve the photocatalytic efficiency. However, most of the metal oxides or metal sulfides are, due to their chemical nature, not durable enough in corrosive media, such as acidic, alkaline, reducing, oxidizing or chelating conditions which are frequently encountered in real wastewater samples. Stability of the applied catalysts is pivotal in real wastewater treatment, as the decomposition or leaching not only results in cost rising but also polluting the water for treatment. At present, little work concerning the catalyst leaching was reported. When the photogenerated electrons (e^−^) are captured by the heavy metal ions, the holes (h^+^) will inevitably accumulate in the valence band. Although the band potential of g-C_3_N_4_ (*E*_VB_ ∼ 1.5 V) is higher than that of oxygen (O_2_), (2H_2_O − 4e^−^ → O_2_ + 4H^+^, *ϕ*_O_2_/H_2_O_ = 1.23 V), it is not positive enough to oxidize water to O_2_ due to the large kinetic barrier in the four-electron oxygen evolution reaction (OER), which makes OER in conventional g-C_3_N_4_–metal oxide heterojuncters particularly sluggish. If the accumulated h^+^ were not effectively removed by water molecule, they would oxidize the catalyst itself, leading to the g-C_3_N_4_ break down.^10^ Although g-C_3_N_4_ can be protected by using sacrificial electron donor (usually organic additives such as alcohols^10^), such a strategy works well only when the real wastewater contains both Cr(vi) and organic pollutants. As some Cr(vi) wastewater contains organic pollutants and some not, searching efficient and stable heterostructure-forming units that can not only enhance the separation of photogenerated charge carriers but also promote OER during g-C_3_N_4_ photocatalytic reduction is of paramount importance. In this regard, ruthenium oxide (RuO_2_) is with no doubt an appealing material for forming heterostructures due to its superior chemical stability,^11,12^ metallic conductivity [2.0–2.5 × 10^4^ S cm^−1^], and high catalytic activity for the OER^13^ for its optimal oxygen binding energy. In addition, the precursor of RuO_2_ is relatively lower cost as compared to other precious metals. RuO_2_ nanoparticles-accommodated g-C_3_N_4_ for photocatalytic oxidation of trichloroethylene has been reported.^14^ However, its action in promoting photocatalytic reductions is rarely reported. In this report, effect and mechanism of RuO_2_ on promoting the g-C_3_N_4_ photocatalytic Cr(vi) reduction was examined and explored. It was found that small amount of RuO_2_ deposition on g-C_3_N_4_ can improve its photocatalytic reduction activity greatly, even without the addition of extra organic additives. The composite is stable in the reaction but also protects the g-C_3_N_4_ from decomposition, which means that the RuO_2_ has multiple effect on the photocatalytic ability and greatly broaden the application of RuO_2_/g-C_3_N_4_ composite in wastewater treatment.

## 2. Experimental

### 2.1. Materials

Ruthenium chloride (RuCl_3_·3H_2_O) and melamine (C_3_H_6_N_6_, 99%) were purchased from Tianjin Meiske Chemical Co., Ltd and Tianjin Zhiyuan Chemical Reagent Co., Ltd respectively. Potassium dichromate (K_2_Cr_2_O_7_), methanol (CH_3_OH), 1,5-diphenylcarbazide, acetone (CH_3_COCH_3_) and *tert*-butanol ((CH_3_)_3_COH) were all of analytical reagent grade and used as received. Ultra-pure water for preparation of any solution was obtained from the Milli-Q® system.

### 2.2. Syntheses of g-C_3_N_4_ and RuO_2_/g-C_3_N_4_ composite

g-C_3_N_4_ was prepared by calcining melamine in a muffle furnace.^15^ Typically, 10 g of melamine was placed into an alumina crucible with a cover and heated from room temperature with a ramp rate of 10 °C min^−1^ to 520 °C and then sustained for 4 h in a muffle furnace. After natural cooling, product in the crucible was transferred into a mortar to be grinded into yellow g-C_3_N_4_ powder (20 min) for later use.

For the synthesis of RuO_2_/g-C_3_N_4_ composite, *in situ* deposition by forced hydrolysis and oxidation of ruthenium chloride was adopted.^11^ Briefly, 0.02 g of RuCl_3_·3H_2_O was dissolved in 30 mL of 60 °C aqueous g-C_3_N_4_ suspension with stirring for 60 min. The obtained mixture was evaporated to get the gel. The resulting gel was dried in an electrothermal drier at 105 °C for 24 h and then was added into an alumina crucible with a cover and heated from room temperature with a ramp rate of 10 °C min^−1^ and then sustained at 520 °C for 30 min. Finally, RuO_2_/g-C_3_N_4_ composite was obtained and labeled as RuO_2_ (*x*%)/g-C_3_N_4_, where “*x*%” denoted the mass fraction of RuO_2_ in the composite.

### 2.3. Characterization

X-ray diffraction (XRD) patterns of the pure g-C_3_N_4_ and RuO_2_/g-C_3_N_4_ composite were examined by a Rigaku D/Max-Ultima^+^ diffractometer equipped with Kα radiation of Cu (*λ* = 0.15418 nm). Transmission electron microscopy (TEM) of g-C_3_N_4_ and RuO_2_/g-C_3_N_4_ were investigated operating at 200 kV through a JEOL JEM-2100 electron microscope. X-ray photoemission spectroscopy (XPS) analysis was evaluated in a Kratos-AXIS ULTRA DLD equipped with a monochromatic Al Kα X-ray source. UV-Vis diffuse reflectance spectra of g-C_3_N_4_ and RuO_2_/g-C_3_N_4_ were analyzed on a TU-1901 UV/Vis spectrophotometer with an IS19-1 integrating sphere to collect the diffusing light with BaSO_4_ as the reference. FTIR spectra of the RuO_2_/g-C_3_N_4_ KBr mixed disks, were recorded utilizing a Thermo Nicolet is5 (400 to 4000 cm^−1^). PL spectra were recorded by RF-5301 PC spectrophotometer using a 150 W xenon lamp at *λ* ∼ 365 nm.

### 2.4. Photocatalytic reduction of aqueous Cr(vi)

**Fig. 1:**
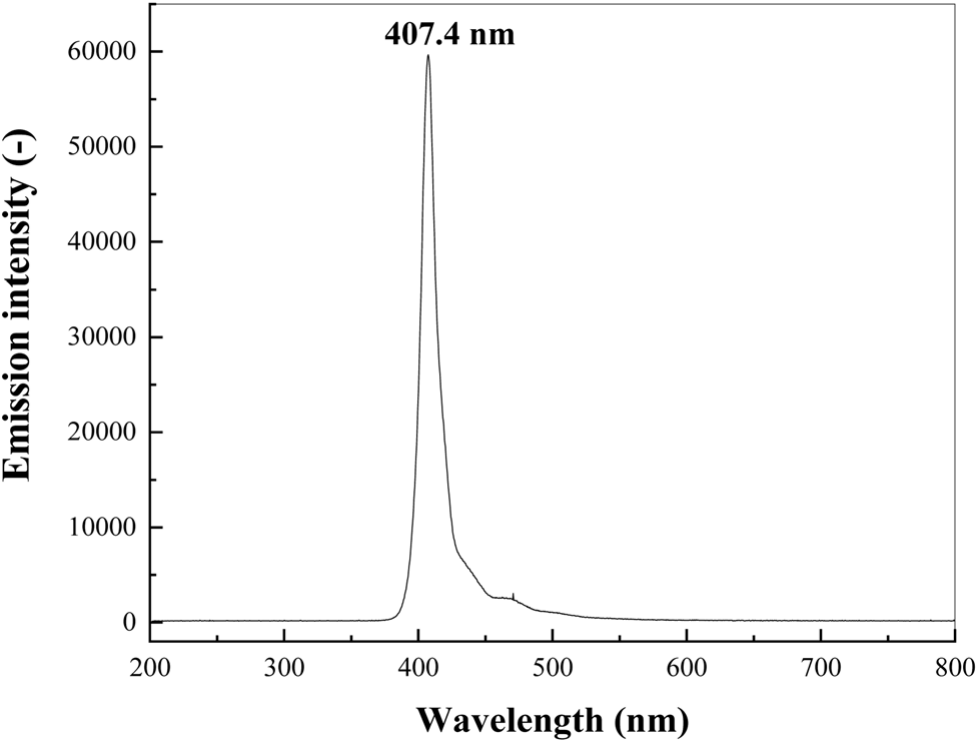
Emission spectra of the LED lamp used for the photocatalytic reduction of Cr(vi).

The photocatalytic reduction activity of g-C_3_N_4_ and RuO_2_/g-C_3_N_4_ composite was tested for Cr(vi) reduction in an aqueous solution. In each typical run, 0.1 g of the prepared photocatalyst was added to 200 mL of 0.5 mM Cr(vi) solution. The mixture was magnetically stirred without illumination for 30 min to attain the adsorption–desorption equilibrium. The suspension was then illuminated by an LED lamp (40 W) surrounded outside the reactor with wavelength centered at 407.4 nm with full-width at half-maximum (FWHM) of 24.7 nm for 2 h with intensity of 25 mW cm^−2^, where the emission spectra of the LED lamp was presented in Fig. 1.

During the illumination, 4.0 mL of the suspension was taken out from the reactor every 15 min, followed by filtration with 0.22 μm membrane and then the filtrate was subjected to chemical analyses. Cr(vi) concentration in the filtrate was determined spectrophotometrically at 520 nm where 1,5-diphenylcarbazide was used as the coloration developer. The photocatalytic reduction efficiency (*η*) of the prepared catalysts was determined as:1
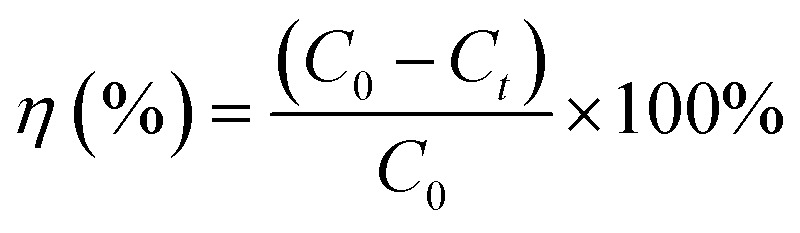
where *C*_0_ and *C*_*t*_ is the Cr(vi) concentration at the beginning of illumination and illumination time *t* (min), respectively. Possible ruthenium leaching from the catalyst was analyzed by an inductively coupled plasma atomic emission spectrometer (ICPAES, Prodigy, Leeman Laboratories). The residue catalysts were evaluated by TOC analysis of the filtrate. Oxygen and nitrogen evolved during the photocatalytic reactions were collected by an air bag and analyzed with a gas analyzer (ST8100A, Smart Sensor Co. Ltd, China).

## 3. Results and discussions

### 3.1. Catalyst characterizations

**Fig. 2:**
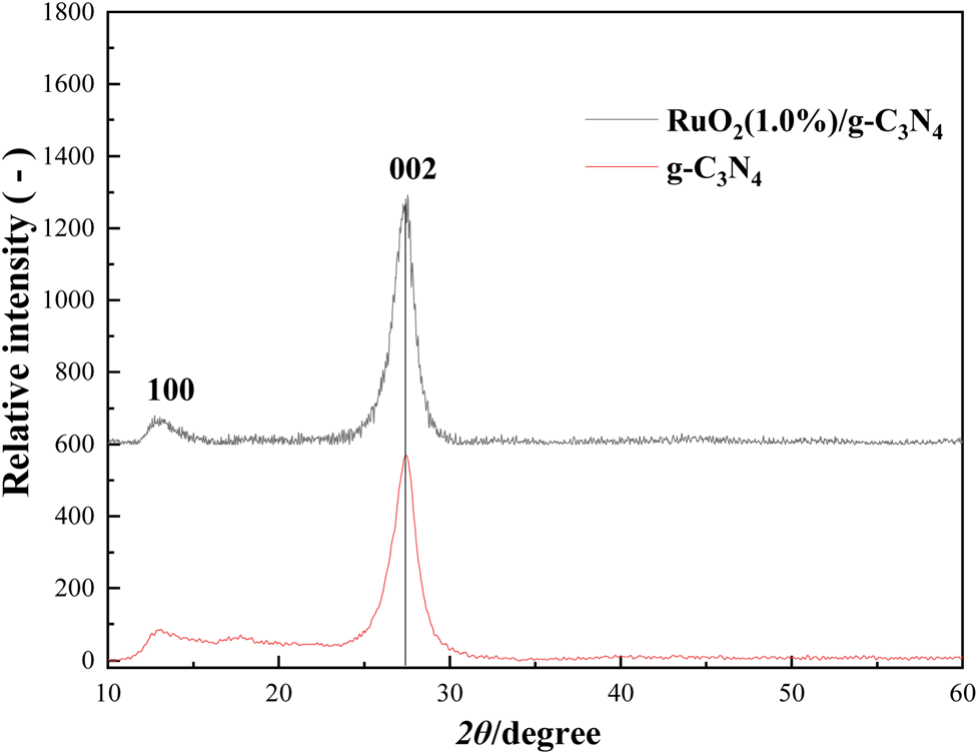
XRD patterns of bare g-C_3_N_4_ and RuO_2_ (1.0%)/g-C_3_N_4_ composite.

Fig. 2 shows the XRD patterns of the prepared bare g-C_3_N_4_ and RuO_2_/g-C_3_N_4_ composite, respectively.

As shown in Fig. 2, two distinct diffraction peaks were observed for pure g-C_3_N_4_. The weak low-angle reflection peak at 12.74° (*d*_100_ = 0.694 nm) was originated from in-planar repeating of tri-*s*-triazine (melem) unit and the peak centered at 27.42° was attributed to the periodic interlayer-stacking (*d*_002_ = 0.325 nm) of the polymeric melon, implying successful condensation of melamine and the distinctive graphitic structure of C_3_N_4_ formed.^4^ XRD pattern of RuO_2_ (1.0%)/g-C_3_N_4_ composite was quite similar to that of bare g-C_3_N_4_, indicating that the composition and structure of g-C_3_N_4_ was not altered appreciably with deposition of the RuO_2_. In addition, no peaks for RuO_2_ were observed, possibly because of its low contents.^14^

According to the Scherrer's formula, the thickness (*D*_002_, nm) of bare g-C_3_N_4_ and RuO_2_ (1.0%)/g-C_3_N_4_ composite can be estimated by:2
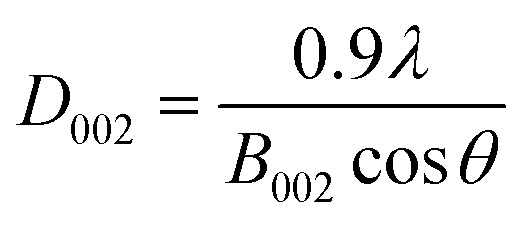
where *λ* is wavelength of the X-ray used for diffraction (nm), *B*_002_ is the FWHM of the diffraction peak at 27.42° (rad) and *θ* is the Bragg angle (°). It can be calculated that the thicknesses of g-C_3_N_4_ and RuO_2_ (1.0%)/g-C_3_N_4_ were about 5.9 nm and 5.7 nm, respectively. As the interlayer-stacking of g-C_3_N_4_ is *ca.* 0.325 nm, the sheet of the above catalysts consists of 18–19 layers of polymeric melons.^15^ Nanosheets usually possess high conductivity, which is beneficial to the charge transfer.^16^

**Fig. 3:**
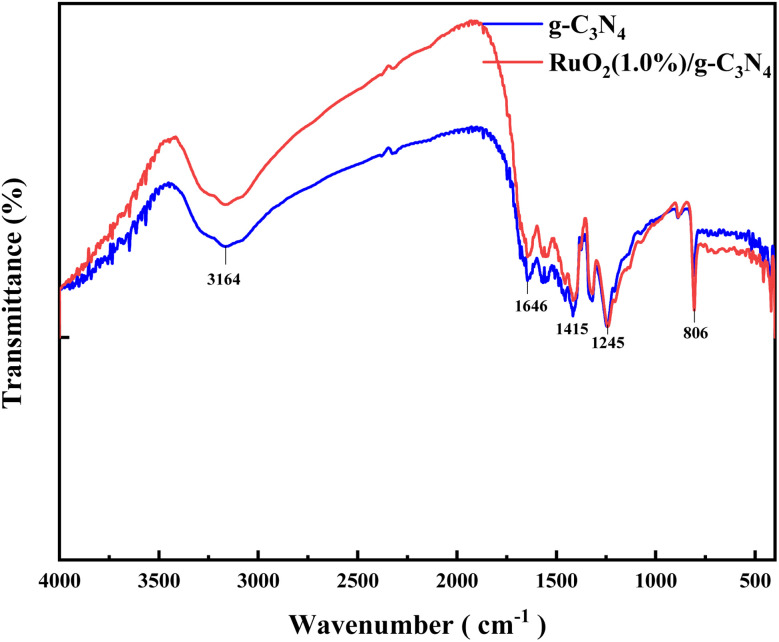
FTIR spectra of bare g-C_3_N_4_ and RuO_2_ (1.0%)/g-C_3_N_4_ composite.

FTIR spectra of bare g-C_3_N_4_ and RuO_2_ (1.0%)/g-C_3_N_4_ composite were determined as shown in Fig. 3.

The peak at 806 cm^−1^ is originated to the breathing of the heptazine ring system. The absorption bands between 1200 and 1700 cm^−1^ indicate the presence of C–N heterocycles. The broad peak at 3164 cm^−1^ was due to the stretching of the terminal N–H from the uncondensed amine.^14^ FTIR spectra of RuO_2_/g-C_3_N_4_ composite are comparable to those of bare g-C_3_N_4_ in the lower wavenumber range. However, the peak intensities of RuO_2_/g-C_3_N_4_ composite were a little weaker than those of bare g-C_3_N_4_,^15^ indicating loss of N–H bond during the preparation of RuO_2_/g-C_3_N_4_ composite.

**Fig. 4:**
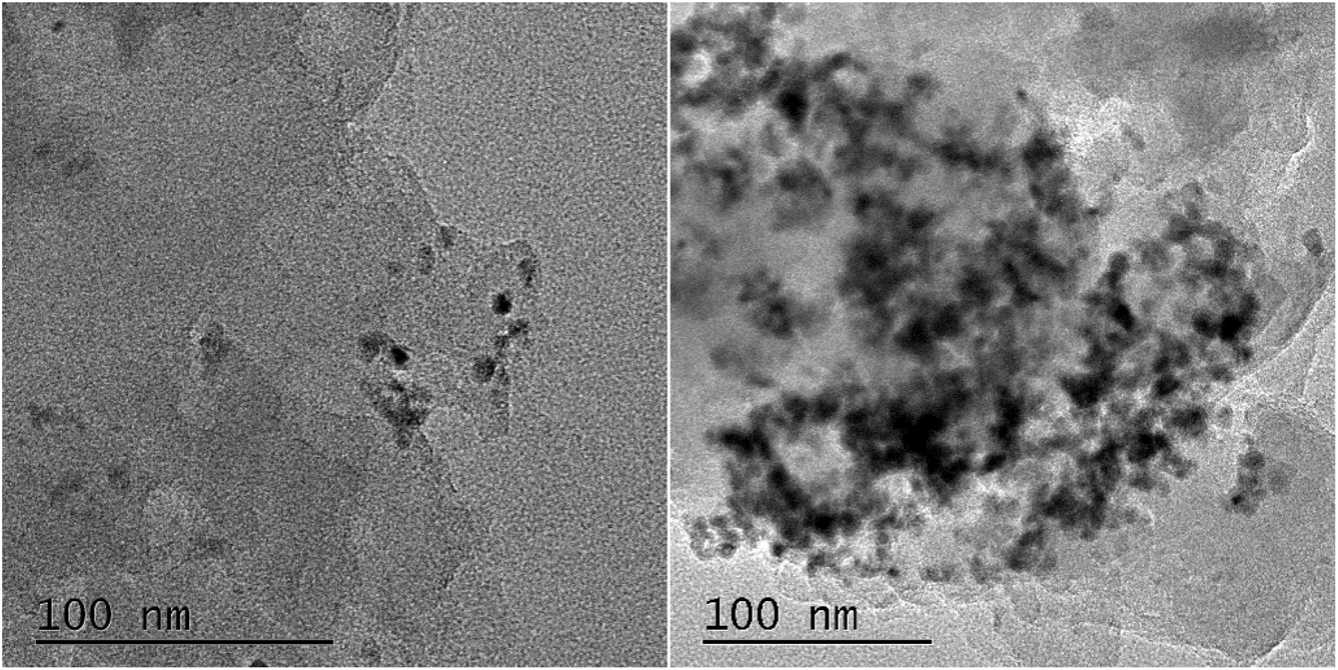
TEM images of pure g-C_3_N_4_ (left) and RuO_2_ (1.0%)/g-C_3_N_4_ composite (right).

Representative TEM images of bare g-C_3_N_4_ and RuO_2_ (1.0%)/g-C_3_N_4_ composite were presented in Fig. 4a and b respectively.

**Fig. 5:**
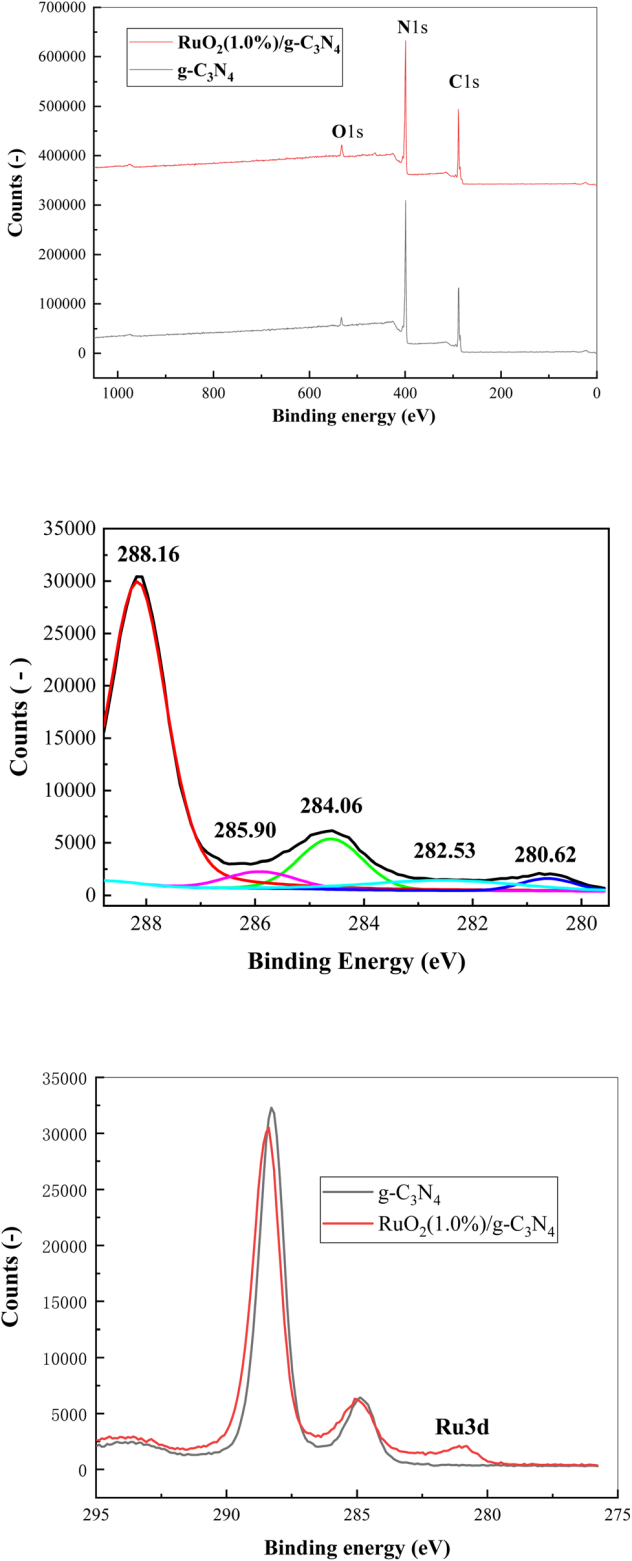
XPS survey spectra of g-C_3_N_4_ and RuO_2_ (1.0%)/g-C_3_N_4_ (upper), high resolution XPS of RuO_2_ (1.0%)/g-C_3_N_4_ (278–294 eV, middle) and Ru 3d scan of RuO_2_ (1.0%)/g-C_3_N_4_ (lower).

The TEM image of pure g-C_3_N_4_ indicated a wrinkled-layer structure along with some stacking layers that have a thin sheet and a typical lamellar morphology. The TEM image of RuO_2_ (1.0%)/g-C_3_N_4_ composite revealed that small RuO_2_ particles are evenly dispersed on the g-C_3_N_4_ surface. The size of RuO_2_ particles is in the range 10–20 nm. HRTEM images and STEM-EDS mapping of RuO_2_ (1.0%)/g-C_3_N_4_ composite were presented in Fig. S1 and S2,† respectively. It can be clearly observed from Fig. S1† that the lattice fringe of RuO_2_ was present in the sample. Fig. S2† indicated the distribution of Ru agrees well with those of both dark filed and bright field mapping but differs slightly from that of oxygen, meaning that the oxygen not only comes from RuO_2_ but also from g-C_3_N_4_ (Fig. 5). These results verified that RuO_2_ is tightly contacted with g-C_3_N_4_, forming the RuO_2_/g-C_3_N_4_ heterojuncters.

To further identify the chemical structure of g-C_3_N_4_ and RuO_2_/g-C_3_N_4_, XPS analyses were performed and given in Fig. 5. The XPS survey spectra of g-C_3_N_4_ confirm that g-C_3_N_4_ is mainly composed of carbon and nitrogen, with a small amount of oxygen. The existence of oxygen in g-C_3_N_4_ is likely due to oxidation during the condensation polymerization. It is noteworthy that the relative intensity of nitrogen peak in g-C_3_N_4_ is higher than that in RuO_2_/g-C_3_N_4_. The higher nitrogen content in g-C_3_N_4_ can be attributed to the preservation of –NH_2_ and NH groups. The peaks located at 280.62 eV and 282.53 eV belong to 3d_5/2_ of RuO_2_.^14^ The theoretical peaks of Ru 3d_3/2_ should be observed at 284.88 eV and 286.78 eV, however as they are partially overlapped with those from C 1s, making it difficult to differentiate them, which is consistent with the results of Hwang *et al.*^17^ RuO_2_ exists in Ru^4+^ state in RuO_2_ (1.0%)/g-C_3_N_4_, indicating the successful deposition of RuO_2_ on the g-C_3_N_4_ surface.

**Fig. 6:**
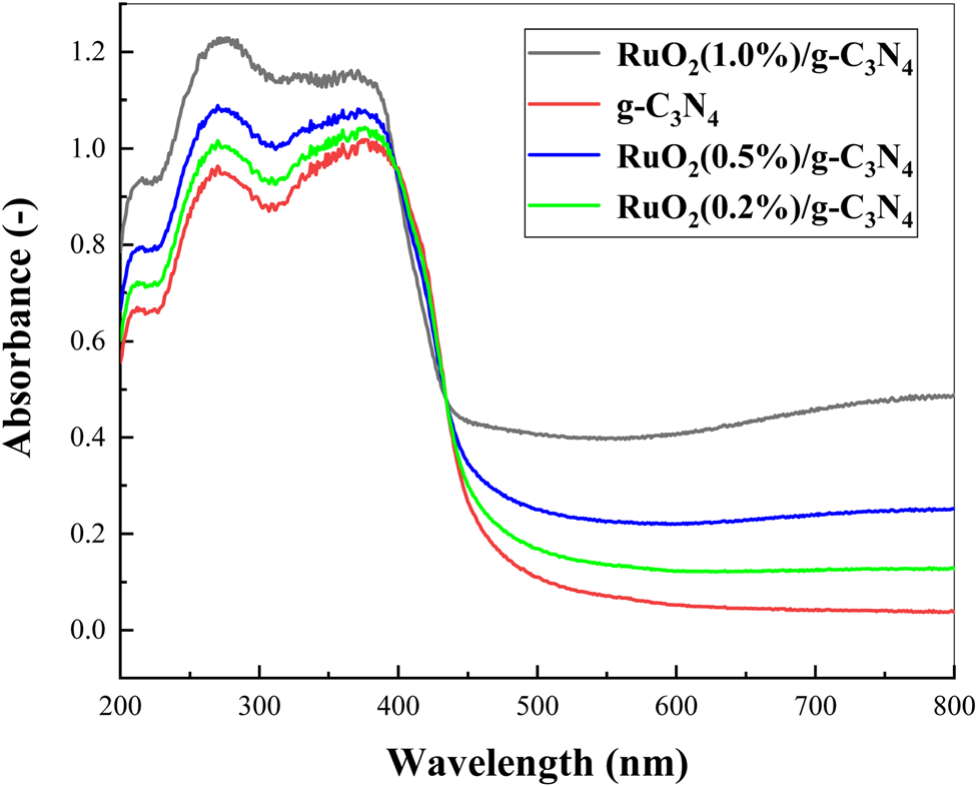
UV-Vis absorption spectra of pure g-C_3_N_4_ and RuO_2_/g-C_3_N_4_ composite.

UV-Vis absorption spectra of the pure g-C_3_N_4_ and the RuO_2_/g-C_3_N_4_ composite at various concentrations were illustrated in Fig. 6. It can be observed from Fig. 6 that all of them were capable of visible light absorption. In general, absorption of the RuO_2_/g-C_3_N_4_ composite was stronger than the bare g-C_3_N_4_, especially in the visible range. However, the absorption of RuO_2_/g-C_3_N_4_ composite was weaker than bare g-C_3_N_4_ in the range 397–434 nm, which is different from those reported earlier.^14^ Outside the wavelength range, the absorption decreases with increasing RuO_2_ loading.^15,16^

### 3.2. Photocatalytic reduction of Cr(vi) by g-C_3_N_4_ and RuO_2_/g-C_3_N_4_

**Fig. 7:**
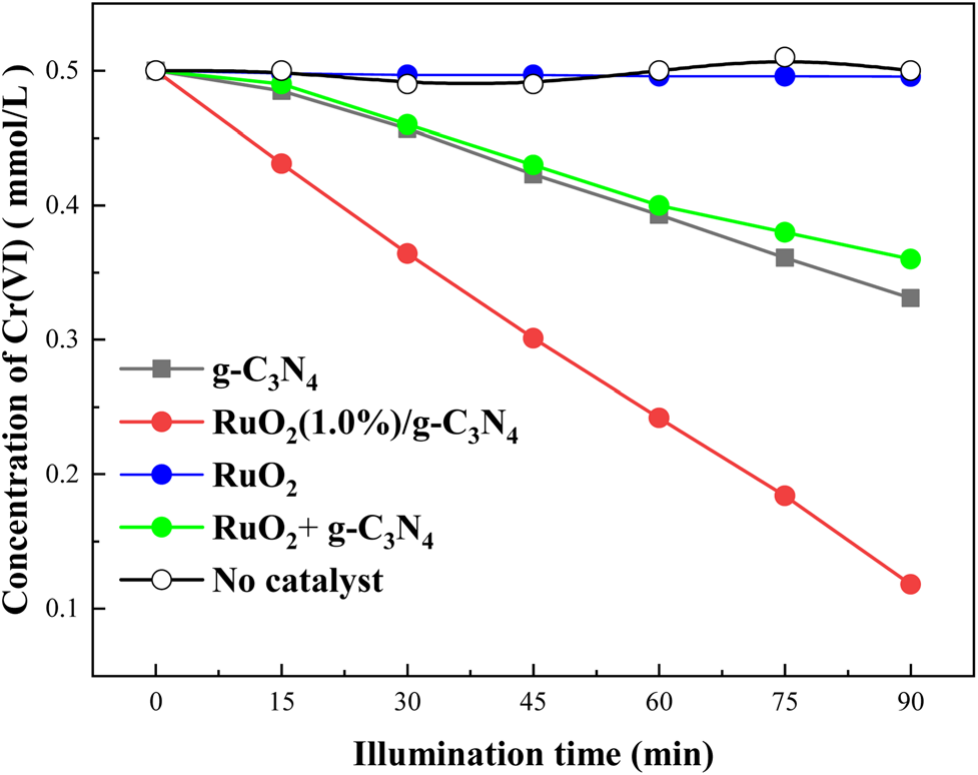
Photocatalytic reductions of Cr(vi) by pure g-C_3_N_4_, pure RuO_2_ and RuO_2_ (1.0%)/g-C_3_N_4_ composite (solution volume, 200 mL; catalyst, 0.1 g; initial Cr(vi) concentration, 0.5 mM; initial pH, 2.3; light source, LED (410 nm, 40 W)).

Variations in Cr(vi) concentration with illumination time in the presence of 0.1 g of pure g-C_3_N_4_, pure RuO_2_ and RuO_2_ (1.0)/g-C_3_N_4_ composite were shown in Fig. 7, respectively.

As seen in Fig. 7, Cr(vi) concentration decreased smoothly with the illumination time. However, the Cr(vi) concentration decreased much more rapidly in the case of RuO_2_/g-C_3_N_4_ composite than pure g-C_3_N_4_ or RuO_2_. After 90 min of illumination, the Cr(vi) removal is *ca.* 76.4% for RuO_2_ (1.0%)/g-C_3_N_4_ and 33.8% for pure g-C_3_N_4_, while it is negligible for pure RuO_2_ (<5%). The Cr(vi) reduction rate (calculated at the initial stage) with RuO_2_ (1.0%)/g-C_3_N_4_ composite is 4.6 times that of the g-C_3_N_4_, which obviously confirms the promoting effect of RuO_2_ in photocatalytic reduction activity of g-C_3_N_4_. It is noted that the Cr(vi) removal is much less when the combination of RuO_2_ and g-C_3_N_4_ was used to reduce Cr(vi), illustrating the successful preparation of heterojunction. In addition, Fig. 7 also showed that the Cr(vi) removal due to adsorption and the direct photolysis were both negligible.^18,19^ Therefore, the present experiments do not consider the direct photolysis of Cr(vi) in the subsequent experiments. When the illumination time is increased to 150 min, the Cr(vi) is below the detection limit (not shown in the figure) in the case of RuO_2_ (1.0%)/g-C_3_N_4_, which means that Cr(vi) can be totally reduced without any sacrificial electron donor.

### 3.3. Effect of initial pH on Cr(vi) photocatalytic reduction

**Fig. 8:**
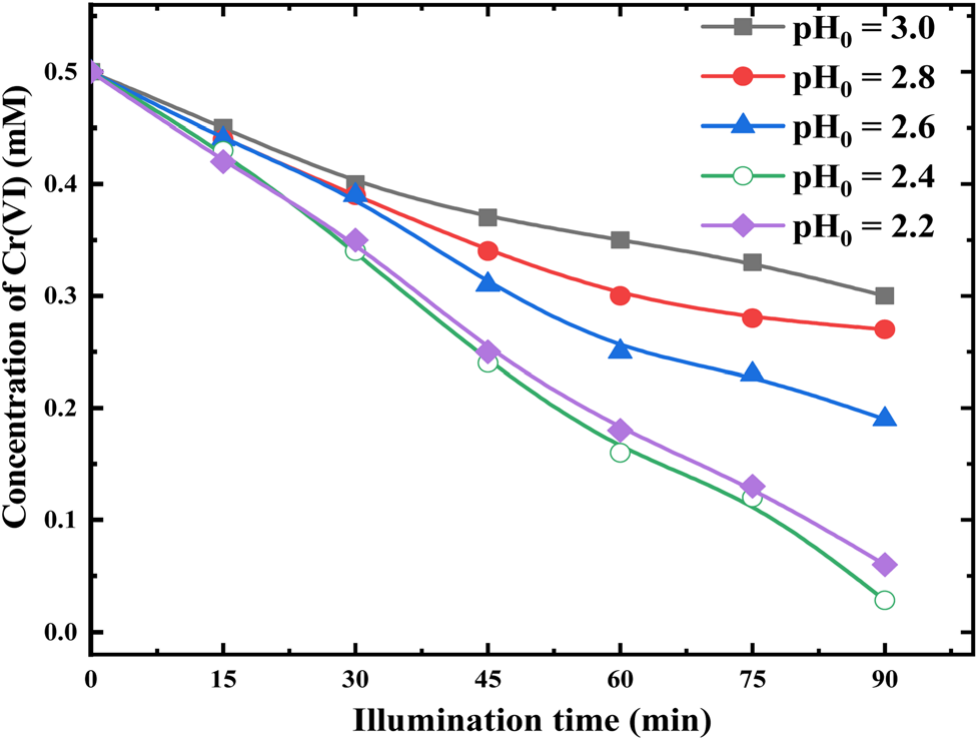
Photocatalytic reduction of Cr(vi) with RuO_2_ (1.0%)/g-C_3_N_4_ under different pH_0_ values (solution volume, 200 mL; catalyst, 0.1 g; initial Cr(vi) concentration, 0.5 mM; light source, LED (410 nm, 40 W)).

The natural pH of the Cr(vi) solution prepared by dissolving K_2_Cr_2_O_7_ in ultrapure water is about 5.5, at which little Cr(vi) reduction was observed under photocatalytic conditions. Therefore, we studied Cr(vi) reduction in acidic medium. Fig. 8 shows Cr(vi) photocatalytic reductions in the presence of 0.1 g RuO_2_ (1.0%)/g-C_3_N_4_ composite under different initial pH (pH_0_) values.

It is demonstrated from Fig. 8 that the Cr(vi) reduction proceeds more rapidly in lower pH_0_. However, the trend is reversed at pH_0_ 2.4. After 90 min of illumination, the Cr(vi) removal is 40% at pH_0_ 3.0 and increases to 95% at pH_0_ 2.4 and drops to 88% at pH_0_ 2.2. The above phenomena can be explained as follows.

In the present experimental conditions, Cr(vi) exists mainly in the form of dichromate (Cr_2_O_7_^2−^) and hydrogen chromate (HCrO_4_^−^). Their mutual relations can be described by reactions (3) and (4).^20,21^

3 Cr_2_O_7_^2−^ + H_2_O ⇄ 2HCrO_4_^−^*K*_3_ = 0.22 M

4 HCrO_4_^−^ ⇄ CrO_4_^2−^ + H^+^*K*_4_ = 3.2 × 10^−7^ M

Over 96% of Cr(vi) is present in the form HCrO_4_^−^ and *ca.* 4% is in the form Cr_2_O_7_^2−^ within pH range 2.0–5.0 at 0.5 mM Cr(vi). As the reaction between HCrO_4_^−^ and e^−^ is promoted by H^+^ (reaction (5)), the Cr(vi) reduction rate increases as the solution pH decreases.^21^

5 HCrO_4_^−^ + e^−^ + H^+^ → H_2_CrO_4_^−^ (Cr(v))

At high concentrations of H^+^, completing reaction between H^+^ and e^−^ (reaction (6)) prevails, which leads to less e^−^ available for Cr(vi) reduction. As a result, the Cr(vi) reduction rate decreased with further decreasing pH_0_:^8^

6 2H^+^ + 2e^−^ → H_2_

On the other hand, when using sulfuric acid to lower the solution pH, hydrogen sulfate ion will be inevitably formed, and it will react with HCrO_4_^−^ to form CrSO_7_^2−^, which decreased the effective HCrO_4_^−^ concentration and the Cr(vi) reduction would slow down in highly acidic condition (eqn (7)):^21^

7 HCrO_4_^−^ + HSO_4_^−^ ⇄ CrSO_7_^2−^ + H_2_O *K*_5_ = 0.42 M^−1^

**Fig. 9:**
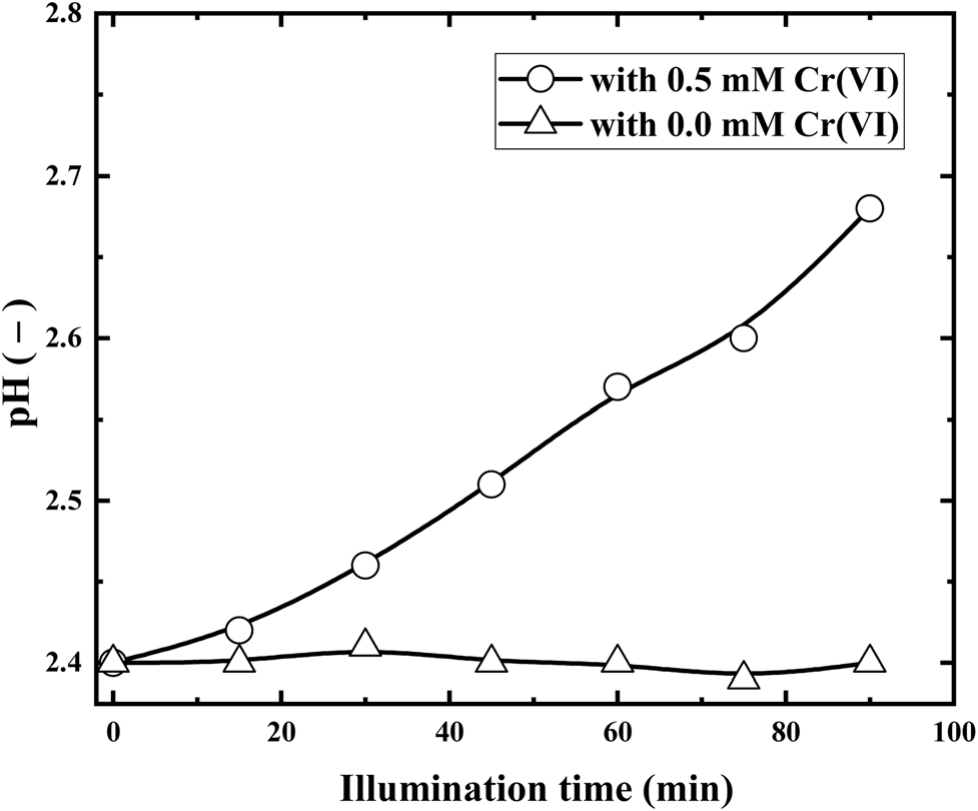
Variations of pH during illumination with and without Cr(vi) (solution volume, 200 mL; RuO_2_ (1.0%)/g-C_3_N_4_ composite, 0.1 g; initial Cr(vi) concentration, 0.5 mM; pH_0_, 2.4; light source, LED (410 nm, 40 W)).

To further elucidate the pH role in Cr(vi) reduction, variations of solution pH during illumination in the presence and absence of Cr(vi) are presented in Fig. 9.

It can be seen from Fig. 9 that the pH of solution containing Cr(vi) increases apparently with illumination time. However, without Cr(vi), pH of the solution changes little. This can be explained by the fact that the Cr(vi) reduction consumes H^+^, as the overall Cr(vi) reduction stoichiometry can be represented by reactions (8) and (9):

8 HCrO_4_^−^ + 3e^−^ + 7H^+^ → Cr^3+^ + 4H_2_O

9
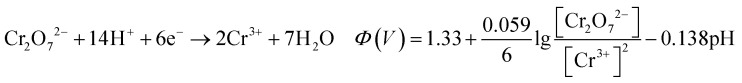


Reactions (8) and (9) need the involvement of H^+^ from the dynamic aspect. As shown in Fig. 8, Cr(vi) reduction proceeded fastest at pH_0_ 2.4, and pH_0_ 2.4 is chosen as the optimum pH in the following investigations.

### 3.4. Effect of electron and hole scavengers on Cr(vi) reduction

**Fig. 10:**
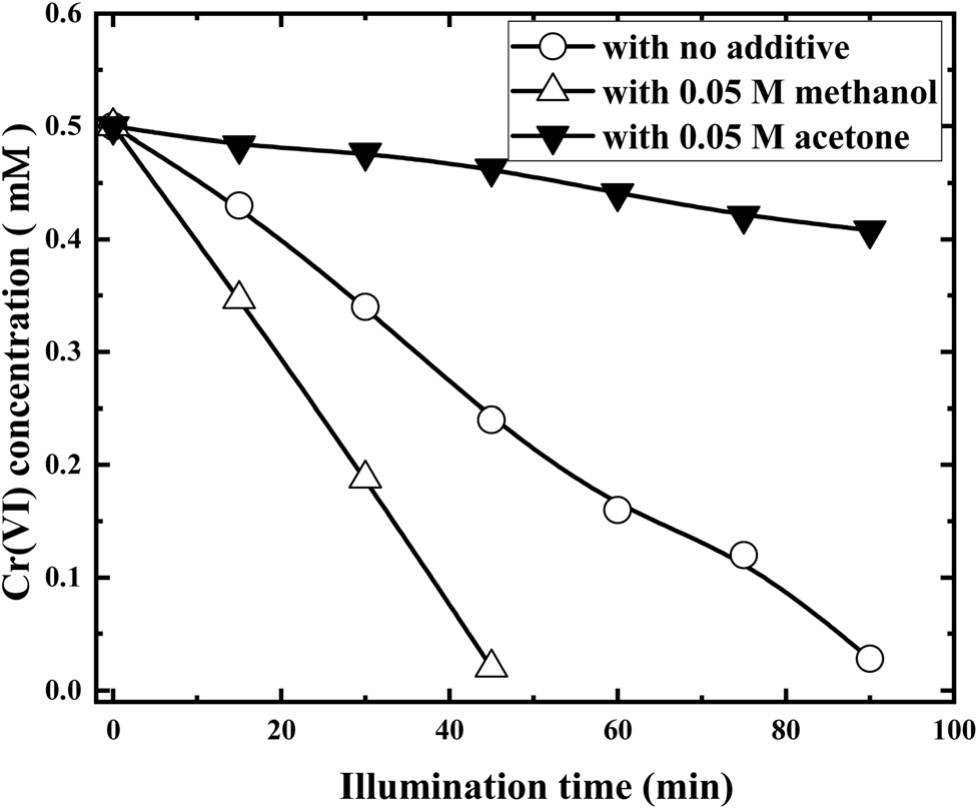
Photocatalytic reductions of Cr(vi) with RuO_2_ (1.0%)/g-C_3_N_4_ in the presence of methanol and acetone (solution volume, 200 mL; RuO_2_ (1.0%)/g-C_3_N_4_ composite, 0.1 g; initial Cr(vi) concentration, 0.5 mM; pH_0_, 2.4; light source, LED (410 nm, 40 W)).

In order to further improve the reduction efficiency of Cr(vi), small amount of organic additives was added to the solution to explore whether the organic solvent will inhibit or promote the reduction of Cr(vi). Fig. 10 shows the Cr(vi) concentration variations in the presence of methanol and acetone, respectively.

It is clearly shown from Fig. 10 that acetone suppresses while methanol promotes the Cr(vi) reduction dramatically. Cr(vi) removal rate was more than doubled and three quarters decreased in the presences of 0.05 M methanol and acetone, respectively. The above phenomena can be explained as follows.

Upon illumination, e^−^ and h^+^ were simultaneously produced. The h^+^ can re-oxidize the Cr(iii) back to Cr(vi) as the oxidation potential of h^+^ (*ca.* 1.5 V) is 0.2 V higher than that of Cr(vi) (*ca.* 1.3 V, reaction (9)). In addition, the h^+^ can recombine with e^−^ to decrease the number of e^−^ available for Cr(vi) reduction. The most desirable way is to convert the h^+^ into organic radicals possessing reduction potentials in the range of −1 to −2 V and are thus capable of reducing Cr(vi) to lower oxidation states, which not only inhibits the re-oxidation of Cr(iii) and increases the concentration of e^−^, but also converts the oxidizing h^+^ to the reducing ones:^22^10
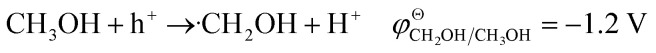


As reaction (10) goes, more e^−^ are available for Cr(vi) reduction. Consequently, the Cr(vi) reduction rate increased in the presence of methanol. On the other hand, as acetone is very stable and cannot be oxidized by the h^+^ but can react with e^−^ as the following:^23–25^11
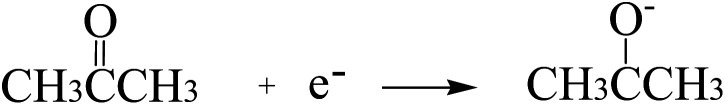


As reaction (11) involved in the process, less e^−^ is available for Cr(vi) reduction and the Cr(vi) reduction rate decreased in the presence of acetone.

### 3.5. Effect of Fe(iii) on Cr(vi) photocatalytic reduction

**Fig. 11:**
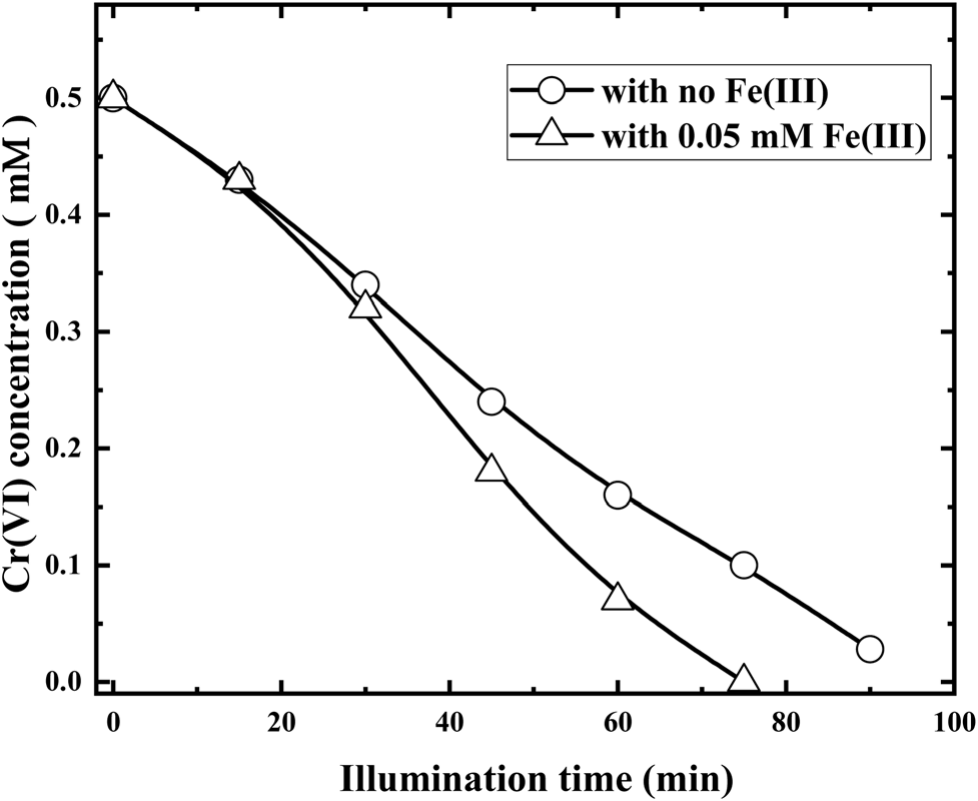
Photocatalytic reductions of Cr(vi) with RuO_2_ (1.0%)/g-C_3_N_4_ in the presence and absence of 0.05 mM Fe(iii) (solution volume, 200 mL; RuO_2_ (1.0%)/g-C_3_N_4_ composite, 0.1 g; initial Cr(vi) concentration, 0.5 mM; pH_0_, 2.4; light source, LED (410 nm, 40 W)).

Fe(iii) is an ubiquitous and nontoxic elements in earth, and investigate its action on photocatalytic reduction of Cr(vi) is of great significance. Fig. 11 compares the Cr(vi) concentration variations in the presence and absence of 0.05 mM Fe(iii), respectively.

It can be clearly observed from Fig. 11 that at the initial stage (<20 min), Fe(iii) displayed little effect on the reduction of Cr(vi). However, the effect becomes apparent at the later stage. The Cr(vi) removal of Cr(vi) at 75 min can reach 100% with 0.05 mM Fe(iii), where it is only 80% without it. When Fe(iii) was added, it was reduced to Fe^2+^ by the e^−^, and then the resulting Fe^2+^ reduces Cr(vi) to Cr(iii) and next circle begins, which means that Fe(iii) can be used as a co-catalyzer.^3^

12 Fe(iii) + e^−^ → Fe^2+^

13 3Fe^2+^ + Cr(vi) → 3Fe(iii) + Cr(iii)

It is noted that as isopropanol ultimately generated by the reaction (11) cannot reduce Cr(vi), the reduction of Cr(vi) is suppressed. Therefore, although both Fe(iii) and acetone belong to electron scavengers, they showed opposing effects on Cr(vi) reductions.

### 3.6. Mechanism of photocatalytic reduction of Cr(vi) by RuO_2_/g-C_3_N_4_

**Fig. 12:**
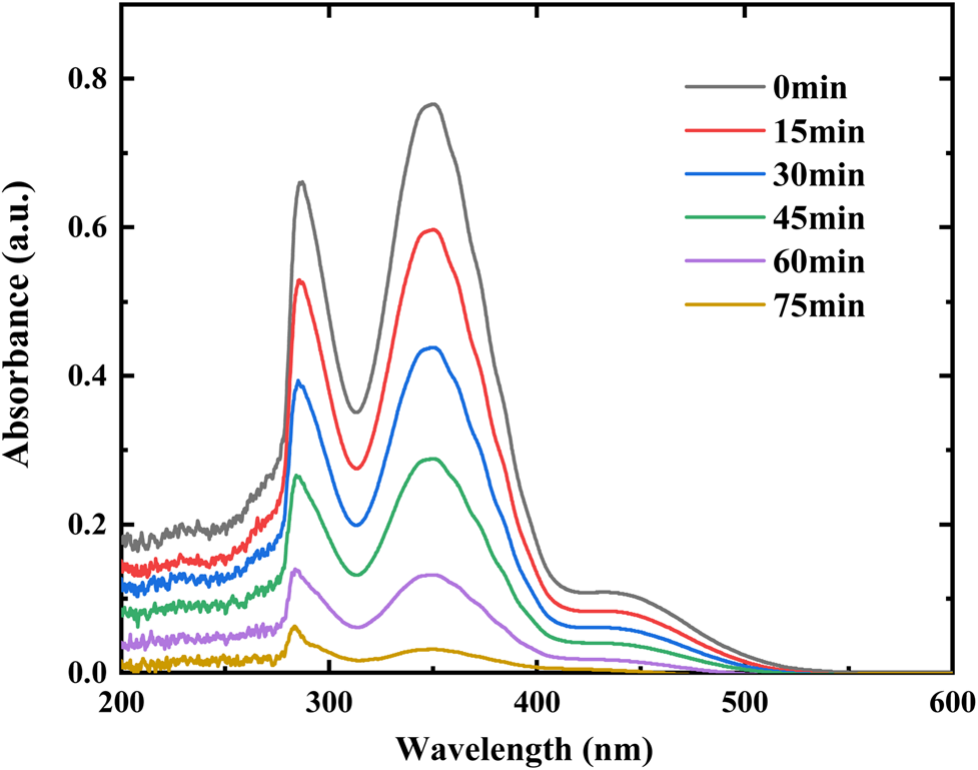
Evolution of UV-Vis absorption spectra of the solution during photocatalytic reduction of Cr(vi) (solution volume, 200 mL; RuO_2_ (1.0%)/g-C_3_N_4_ composite, 0.1 g; initial Cr(vi) concentration, 0.5 mM; pH_0_, 2.4; light source, LED (410 nm, 40 W)).

To elucidate the mechanism in Cr(vi) reduction, evolution of UV-Vis absorption spectra of the solution during photocatalytic reduction are presented in Fig. 12.

As shown in Fig. 12, the peak height at about 350 nm, which is characteristic of the absorption band of Cr(vi) species,^21^ together with that at 285 nm, gradually decreases with the illumination time. In general, Cr(vi) would undergo a series of intermediate processes before being finally converted to Cr(iii). As shown in Fig. 12, no new bands appeared during the reduction, indicating that the possible Cr(iv) and Cr(iv) were too short-lived to be detected by the present technique.^26,27^ In the experiment, color of the solution changed from orange to light yellow and finally to colorless, which was verified by the decrease in absorption band from 400 to 450 nm.

According to ref. 14, g-C_3_N_4_ and RuO_2_ are both n-type semiconductors. The values of *E*_CB_ and *E*_VB_ for pure g-C_3_N_4_ are −1.125 eV and +1.585 eV,^28^ respectively. *E*_CB_ and *E*_VB_ of RuO_2_ can be calculated according to the formula (14) and (15):^29^

14 *E*_CB_ = *E*_VB_ − *E*_g_

**Fig. 13:**
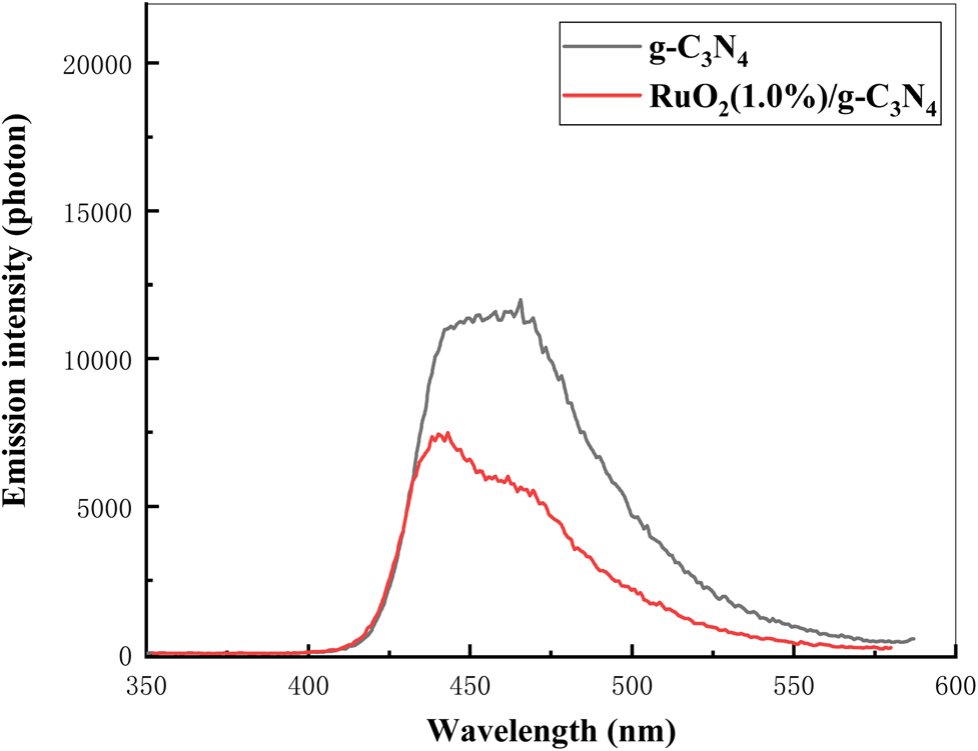
Fluorescence emission spectra of g-C_3_N_4_ and RuO_2_ (1.0%)/g-C_3_N_4_.

15 *E*_VB_ = *X* − *E*_e_ + 0.5*E*_g_

where *X* is the absolute electronegativity of RuO_2_ (6.35 eV),^27^*E*_e_ is the vacuum electron level corresponding to the standard hydrogen (4.5 eV). *E*_g_ of RuO_2_ was determined to be 2.32 eV.^14^ It can be calculated that *E*_CB_ and *E*_CB_ of RuO_2_ are 3.01 eV and 0.69 eV respectively. Because the Fermi level (*E*_F_) of n-type semiconductor is very close to its *E*_CB_, the Fermi levels of g-C_3_N_4_ and RuO_2_ are about −1.125 eV and −0.69 eV. As the *E*_F_ of g-C_3_N_4_ is lower than that of RuO_2_, when g-C_3_N_4_ and RuO_2_ contact each other, the electrons on g-C_3_N_4_ automatically flow to the conduction band of RuO_2_, leaving positive charges, thus forming a built-in electric field between RuO_2_ and g-C_3_N_4_ (g-C_3_N_4_ pointing to RuO_2_). When g-C_3_N_4_ absorbs visible light, electron hole pairs (e^−^ + h^+^) were generated. Under the action of built-in electric field, the e^−^ move towards the solution and are captured by Cr(vi) in the solution, resulting in the reduction of Cr(vi) and suppress the electron hole recombination. Such explanation was documented by Fig. 13.

It can be shown in Fig. 13 that the emission intensity of RuO_2_/g-C_3_N_4_ was much lower than g-C_3_N_4_, showing the suppressed recombination of photo-carriers.

**Fig. 14:**
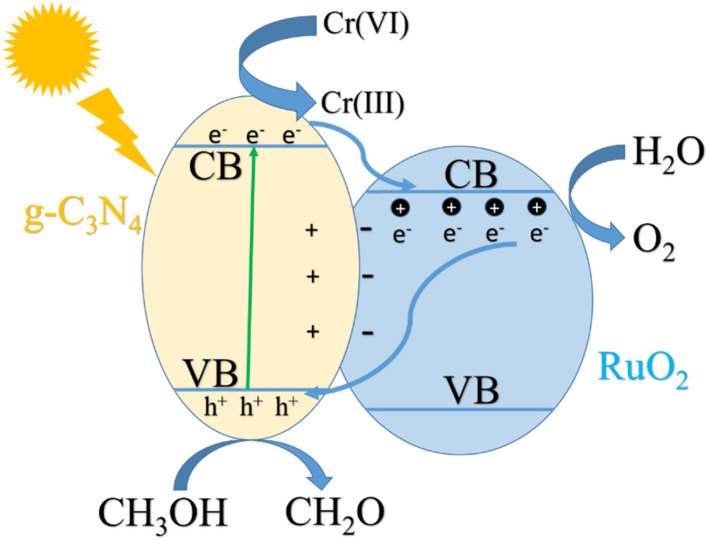
Mechanism of photocatalytic reduction of Cr(vi) by RuO_2_/g-C_3_N_4_.

In addition, the absorption of RuO_2_/g-C_3_N_4_ is slightly weaker than pure g-C_3_N_4_ near 410 nm as shown in Fig. 6, indicating that the role of RuO_2_ belongs to the enhancement of charge separation rather than the enhancement of light absorption, which also indicates that the mechanism speculation is reasonable. The process can be demonstrated in Fig. 14:

On the other hand, the loaded RuO_2_ is able to take up the h^+^ from g-C_3_N_4_, exhibiting functionality as efficient O_2_ evolution sites. The reaction sequence for O_2_ evolution can be expressed as follows:^30^

16 RuO_2_ + 2h^+^ → RuO_2_^2+^

17 RuO_2_^2+^ + H_2_O → RuO_3_ + 2H^+^

18 2RuO_3_ → 2RuO_2_ + O_2_(g)

**Fig. 15:**
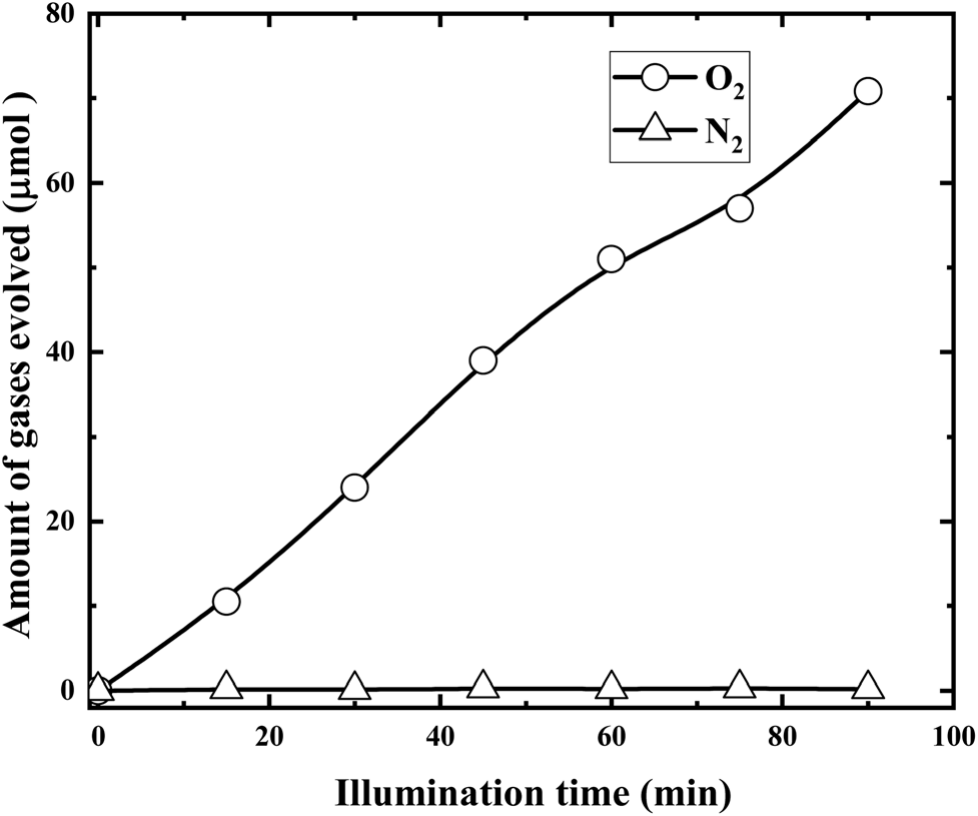
Time courses of O_2_ and N_2_ evolution during the illumination (solution volume, 200 mL; RuO_2_ (1.0%)/g-C_3_N_4_ composite, 0.1 g; initial Cr(vi) concentration, 0.5 mM; pH_0_, 2.4; light source, LED (410 nm, 40 W)).

In order to confirm the above assumptions, time courses of O_2_ and N_2_ evolution during the illumination were examined and the results are presented in Fig. 15.

It is demonstrated from Fig. 15 that RuO_2_ (1.0%)/g-C_3_N_4_ composite exhibited high activity for O_2_ evolution with no N_2_ evolution. This further indicated that RuO_2_ loading not only boost the separation of the charge carriers but also protects the decomposition of g-C_3_N_4_.

### 3.7. Recyclability and stability test

**Fig. 16:**
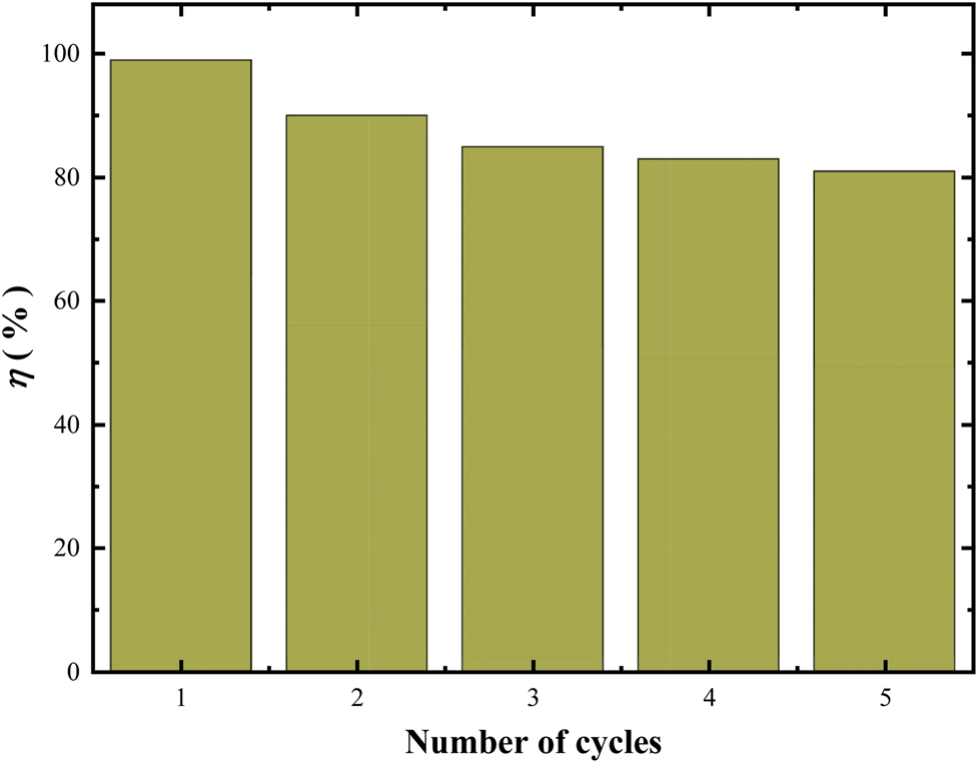
Cycle stability test of RuO_2_ (1.0%)/g-C_3_N_4_ composite (solution volume, 200 mL; RuO_2_ (1.0%)/g-C_3_N_4_ composite, 0.1 g; initial Cr(vi) concentration, 0.5 mM; pH_0_, 2.4; light source, LED (410 nm, 40 W)).

To evaluate the reusability and stability of the samples, five successive cycles of experiments were performed for Cr(vi) reduction. After completion of each reduction reaction (90 min), the catalyst in the solution was filtered with 0.22 μm filter membrane, washed with ethanol and water for 3 times respectively, put it into the oven, and bake at 60 °C for 24 hours for the next experiment. Namely, the used sample after each cycle was collected through centrifugation and rinsed three times with ethanol and ultrapure water. Subsequently, the washed powder was dried for 8 h (60 °C) to conduct next photocatalytic cycle. Moreover, the XRD of the samples after six cycles was characterized and compared to that of the fresh samples. A total of 5 cycles were carried out. The reduction ratio of Cr(vi) at each final cycle is shown in Fig. 16.

It can be seen from Fig. 16 that the Cr(vi) reduction ratio with recycled RuO_2_ (1.0%)/g-C_3_N_4_ still maintains more than 80% after five repeated experiments, indicating that the photocatalytic stability of RuO_2_ (1.0%)/g-C_3_N_4_ is still very good and can be reused. ICP-AES test showed no leaching of Ru form RuO_2_ (1.0%)/g-C_3_N_4_. In addition, TOC analysis also showed no apparent carbon and nitrogen increase in the solution, indicating that RuO_2_ (1.0%)/g-C_3_N_4_ is very stable during the photocatalysis.

## 4. Conclusions

RuO_2_/g-C_3_N_4_ composite can be easily prepared by *in situ* forced hydrolysis and oxidation and is able to catalytically photo-reduce Cr(vi) effectively even in the absence of sacrificial electron donor. The optimum initial pH for the reduction is 2.4. Acetone inhibits the reduction and methanol promotes the reduction. In addition, a small amount of Fe(iii) catalyzes the reduction of Cr(vi), especially in the later stage.

The deposited RuO_2_ particles not only functionalized as an effective charge separator for Cr(vi) reduction, but also protects the g-C_3_N_4_ from self-decomposition through catalyzing O_2_ evolution, which is indispensable for the reduction without presence of electron donors. Little leaching and residue remained in the solution further proves its potential application in real wastewater treatment process.

## Data availability

The authors confirm that the data supporting the findings of this study are available within the article.

## Author contributions

Conceptualization, Y. Liu; methodology, Y. Liu; software, Y. Liu; validation, Y. Liu; formal analysis, Y. Liu; investigation, X. Du and Z. Huang; resources, Y. Liu; data curation, Y. Liu; writing—original draft preparation, Y. Liu; writing—review and editing, Y. Liu; visualization, Y. Liu; supervision, Y. Liu; project administration, Y. Liu; funding acquisition, Y. Liu. All authors have read and agreed to the published version of the manuscript.

## Conflicts of interest

The authors declare no conflict of interest.

## Supplementary Material

RA-015-D5RA00883B-s001
