# Supplementary material for: Photocatalytic reduction of aqueous chromium(vi) by RuO2/g-C3N4 composite under visible light irradiation
Source: RSC Adv. 2025 May 20;15(21):16724–33. doi: 10.1039/d5ra00883b (PMC12089977; doi:10.1039/d5ra00883b)
Supplement: RA-015-D5RA00883B-s001 [file RA-015-D5RA00883B-s001.pdf]

Supplementary Information (SI) for RSC Advances.
This journal is © The Royal Society of Chemistry 2025

Supplementary data

Photocatalytical Reduction of Aqueous Chromium (VI) by RuO_2/g-C_3N_4 Composite under Visible Light Irradiation

Yongjun Liu*, Xiaohe Du, Zhiming Huang

College of Environmental Science & Engineering, Dalian Maritime University, Dalian, 116026, P. R. China; Email: lyjglow@dlmu.edu.cn; Tel: 86-411-84725275; Fax: 86-411-84727670

----

* Corresponding author. Email: lyjglow@dlmu.edu.cn (Y. Liu).

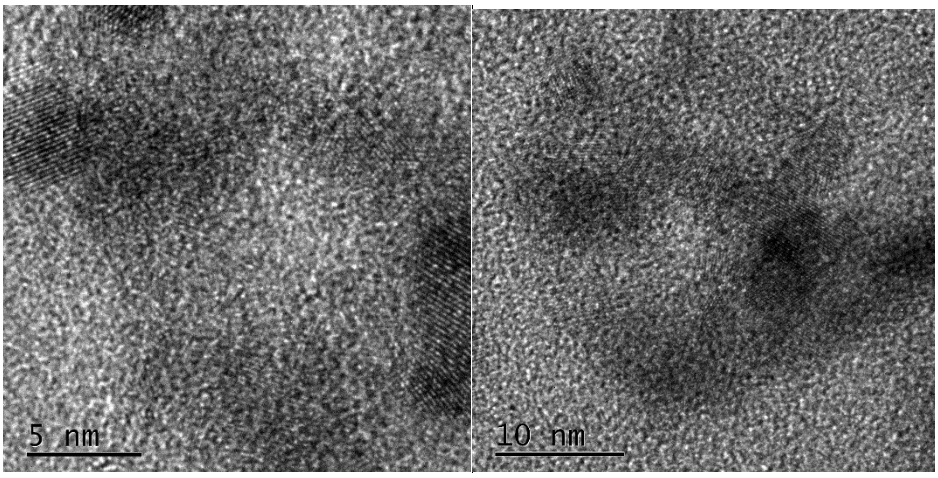

Fig. S1 HRTEM information of RuO_2(1.0%)/g-C_3N_4 composite.

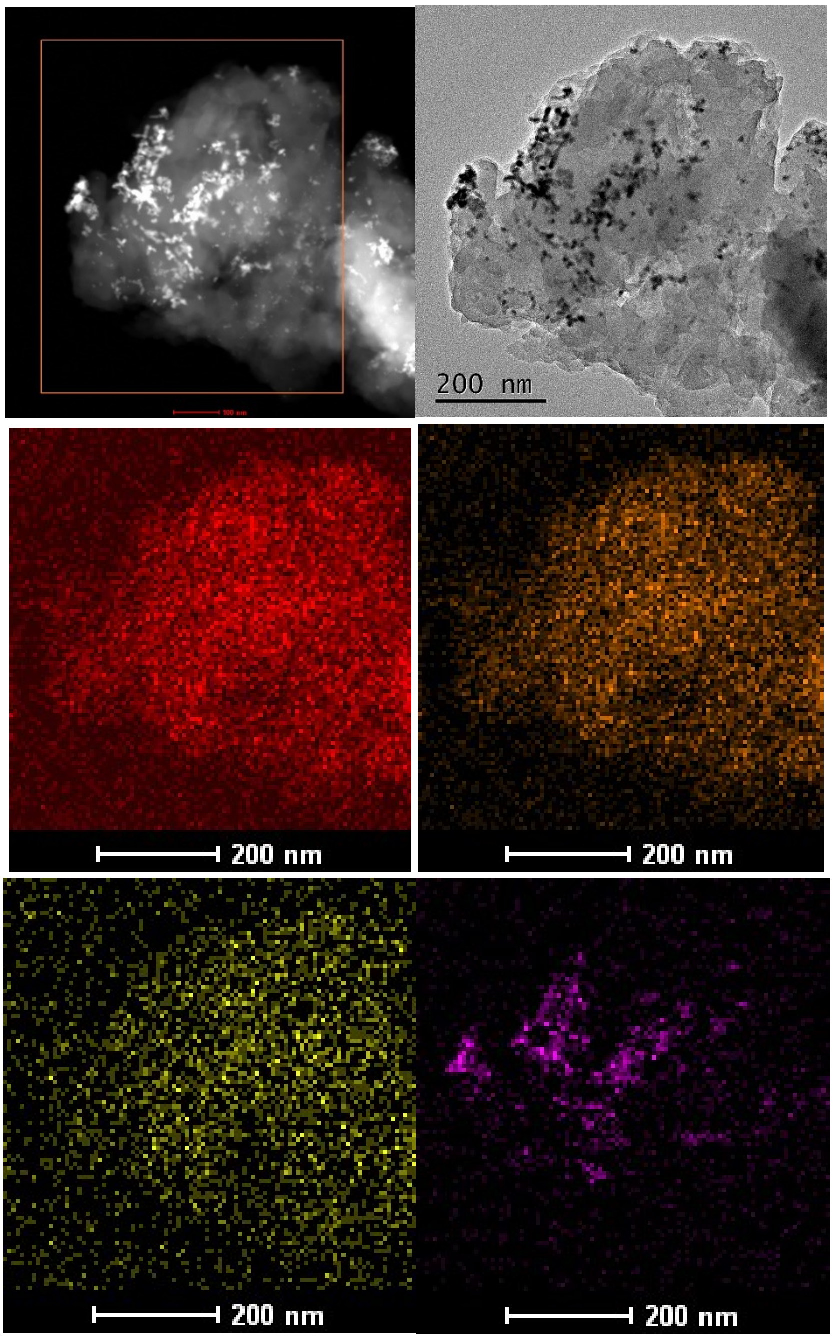

Fig. S2 STEM-EDS mapping of RuO_2(1.0%)/g-C_3N_4 composite (upper left, high angle annular dark field; upper right, bright field; middle left, C-K; middle right, N-K; bottom left, O-K; bottom right, Ru-L).

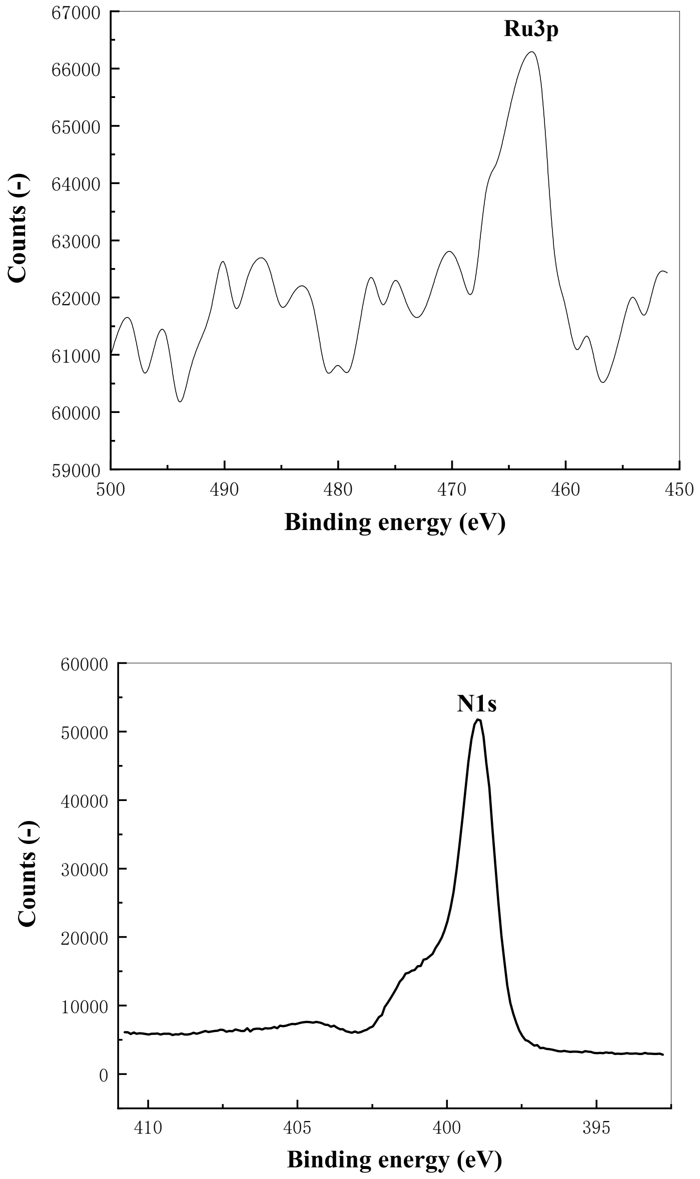

Fig. S3 high-resolution XPS spectra of Ru3p and N1s of RuO_2(1.0%)/g-C_3N_4.
